# Evaluation for Retinal Therapy for *RPE65* Variation Assessed in hiPSC Retinal Pigment Epithelial Cells

**DOI:** 10.1155/2021/4536382

**Published:** 2021-12-13

**Authors:** Benjamin M. Nash, To Ha Loi, Milan Fernando, Amin Sabri, James Robinson, Anson Cheng, Steven S. Eamegdool, Elizabeth Farnsworth, Bruce Bennetts, John R. Grigg, Seo-Kyung Chung, Anai Gonzalez-Cordero, Robyn V. Jamieson

**Affiliations:** ^1^Eye Genetics Research Unit, Sydney Children's Hospitals Network-Westmead, Save Sight Institute, Children's Medical Research Institute, University of Sydney, Sydney, New South Wales, Australia; ^2^Specialty of Genomic Medicine, Faculty of Medicine and Health, University of Sydney, New South Wales, Australia; ^3^Sydney Genome Diagnostics, Western Sydney Genetics Program, Sydney Children's Hospitals Network-Westmead, Sydney, New South Wales, Australia; ^4^Stem Cell Medicine Group and Stem Cell and Organoid Facility, Children's Medical Research Institute, University of Sydney, Faculty of Medicine & Health, Sydney NSW, Australia; ^5^Department of Ophthalmology, Sydney Children's Hospitals Network-Westmead, Sydney, New South Wales, Australia; ^6^Specialty of Ophthalmology, Faculty of Medicine and Health, University of Sydney, Sydney, New South Wales, Australia; ^7^Translational Neurogenomics Group, Kids Research, Sydney Children's Hospitals Network-Westmead, Sydney NSW, Australia; ^8^Brain and Mind Centre, Faculty of Medicine & Health, University of Sydney, Sydney NSW, Australia; ^9^Department of Clinical Genetics, Western Sydney Genetics Program, Sydney Children's Hospitals Network-Westmead, Sydney, New South Wales, Australia

## Abstract

Human induced pluripotent stem cells (hiPSCs) generated from patients and the derivative retinal cells enable the investigation of pathological and novel variants in relevant cell populations. Biallelic pathogenic variants in *RPE65* cause early-onset severe retinal dystrophy (EOSRD) or Leber congenital amaurosis (LCA). Increasingly, regulatory-approved *in vivo RPE65* retinal gene replacement therapy is available for patients with these clinical features, but only if they have biallelic pathological variants and sufficient viable retinal cells. In our cohort of patients, we identified siblings with early-onset severe retinal degeneration where genomic studies revealed compound heterozygous variants in *RPE65*, one a known pathogenic missense variant and the other a novel synonymous variant of uncertain significance. The synonymous variant was suspected to affect RNA splicing. Since RPE65 is very poorly expressed in all tissues except the retinal pigment epithelium (RPE), we generated hiPSC-derived RPE cells from the parental carrier of the synonymous variant. Sequencing of RNA obtained from hiPSC-RPE cells demonstrated heterozygous skipping of *RPE65* exon 2 and the introduction of a premature stop codon in the mRNA. Minigene studies confirmed the splicing aberration. Results from this study led to reclassification of the synonymous variant to a pathogenic variant, providing the affected patients with access to *RPE65* gene replacement therapy.

## 1. Introduction

Leber congenital amaurosis (LCA) and other early-onset severe retinal dystrophies (EOSRD) have a prevalence of approximately 1 : 50,000 worldwide [1]. Symptoms may include nystagmus, photophobia, and the oculodigital sign of eye rubbing or poking, and when these are present in the first year of life, a diagnosis of LCA may be suspected. Ophthalmic investigations demonstrate severely diminished or often undetectable full-field electoretinogram (ffERG) traces, indicating severe photoreceptor pathology [1]. There are currently more than 24 disease genes associated with LCA and other forms of EOSRD and a molecular diagnosis may be obtained in approximately 70-80% of families [2]. In view of the phenotypic and genetic heterogeneity of LCA and EOSRD, the relatively recent mainstream adoption of genomic technologies in diagnostic laboratories has enabled improved access for families to clinical molecular testing and diagnosis.

The *RPE65* gene encodes the retinal-specific isomerohydrolase enzyme, a 533 amino acid, and 61 KDa protein. RPE65 is involved in the visual cycle recovery phase and the conversion of 11-*trans* retinyl ester to 11-*cis* retinal after pigmentary excitation from light [3]. Autosomal recessive homozygous and compound heterozygous disease-causing variants in *RPE65* have been well described and account for ~16% of individuals with LCA or EOSRD, and variants occur less frequently, at ~2%, in individuals with retinitis pigmentosa (OMIM: #18600) [4, 5]. Autosomal recessive LCA and EOSRD-associated *RPE65* variants reported to date include missense, frameshift, premature stop, in-frame deletion, and splicing variants [6]. To date, there are no clear genotype-phenotype correlations reported, with the severity or the onset of disease presentation seemingly independent to the type or location of the causative variants detected [7].

There is a new era emerging in ophthalmology and precision medicine with the first ocular gene therapy receiving regulatory approval in several jurisdictions, namely, the *RPE65* gene replacement therapy voretigene neparvovec-rzyl [8]. To receive this, therapy patients must have clinical features of retinal dystrophy, viable retinal cells, and biallelic pathological *RPE65* gene mutations (https://www.tga.gov.au/apm-summary/luxturna).

Diagnostic genomics is rapidly expanding the ability to efficiently interrogate the human genome, and standardised approaches to variant classification have been adopted widely by diagnostic laboratories [9, 10]. However, sometimes a variant is classified as a variant of uncertain significance (VUS), even if the clinical phenotype is in agreement with the disease. This lack of confirmed pathological diagnosis makes these patients ineligible to receive *RPE65* gene therapy. Functional genomic studies regarding the deleterious effects of a VUS on RNA or protein function are currently considered strong evidence of pathogenicity; however, these studies are often hampered by other technical complexities such as the disease gene being exclusively expressed in clinically unobtainable tissue types.

Advances in stem cell methods and technologies have led to the application of patient-derived hiPSCs to generate retinal pigmented epithelial cells (hiPSC-RPE) and retinal organoids (hiPSC-RO) to model retinal disease mechanisms and therapies directly in target human retinal cells [11, 12]. In this study, we generated a hiPSC-RPE model to examine the effects of a novel genomic VUS in two siblings from a family presenting with autosomal recessive EOSRD. The hiPSC-RPE model facilitated access to *RPE65* mRNA to investigate the molecular consequence of this cryptic genomic variant and determine its pathological impact.

## 2. Subjects and Methods

### 2.1. Human Participants

All human subjects in this study underwent written informed consent. This study was approved by the Human Research Ethics Committee (HREC) of the Sydney Children's Hospital Network, Sydney, Australia, and adhered to the tenets governed by the Declaration of Helsinki.

### 2.2. Ophthalmic Investigations

Detailed ophthalmic review was undertaken and included fundal examination and imaging, ffERG performed according to International Society for Clinical Electrophysiology of Vision (ISCEV) to examine retinal function, wide-field fundus autofluorescence (WF-FAF), and spectral domain optical coherence tomography (SD-OCT) assessment of the retinal structures.

### 2.3. Genomics and Bioinformatics

**Figure 1:**
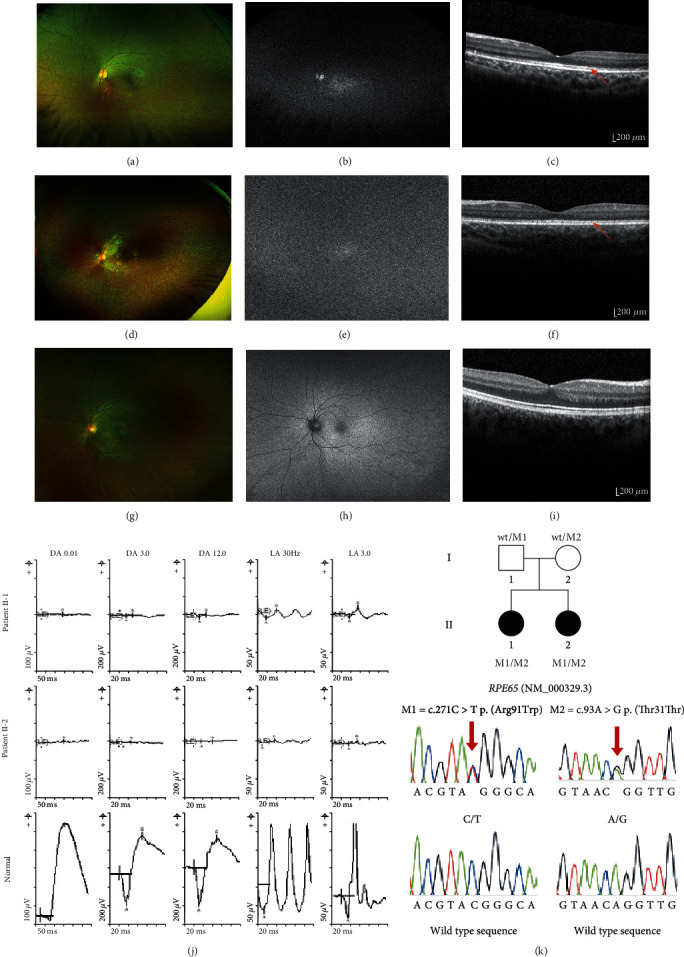
Ophthalmic investigations and genetic findings. Patient II-1: (a) wide-field pseudocolor fundus images showing discrete RPE mottling in the midperiphery illustrating outer retinal atrophy and small white intraretinal spots or flecks in the perimacular region. (b) Wide-field fundus autofluorescence (WF-FAF) showing significantly reduced retinal autofluorescence signal. (c) Optical coherence tomography (OCT) showing photoreceptor complex relatively intact. Patient II-2: (d) wide-field pseudocolor fundus images showing widespread distribution of discrete RPE mottling and white/yellow flecks in the midperipheral fundus. (e) WF-FAF illustrating almost absent fundus autofluorescence. (f) OCT showing preservation of the ellipsoid zone across the macula with slight macular thinning. (g) Normal wide-field pseudocolor fundus image. Note the homogenous retinal background with no white/yellow flecks or blotches (Optos Dunfermline UK). (h) Normal wide-field fundus autofluorescence. Note that the retinal blood vessels and optic disc appear black because they do not fluoresce. Compare this to images (b) and (e) where there is little or no retinal autofluoresence in the whole of the retina so the blood vessels and optic disc merge into the image. (i) Normal OCT scan showing well defined outer retinal structures compared to both patients. (j) Full-field electroretinogram (ffERG) with patient II-1 top row, patient II-2 middle row and age matched normal bottom row. Both patients II-1 and II-2 show undetectable scotopic responses and residual attenuated photopic function. (k) Pedigree showing biparental inheritance of the two *RPE65* alleles and Sanger sequencing traces.

The proband II-1 (Figure 1(k)) underwent commercial targeted capture retinal dystrophy gene panel testing (Molecular Vision Laboratories, USA), examining 280 retinal disease genes. Variant confirmation and segregation studies of candidate variants were undertaken using Sanger sequencing performed by Sydney Genome Diagnostics (Sydney Children's Hospital Network—Westmead, Australia), with PCR products sequenced at the Australian Genome Research Facility (Westmead, Australia). Variants were classified in accordance with established ACMG guidelines [9, 10].

### 2.4. Reprogramming Somatic Cells into hiPSCs

Peripheral blood mononuclear cells (PBMCs) were isolated from a known heterozygous parental carrier of the novel synonymous variant under investigation (Figure 1(k), individual I-2) using Ficoll density centrifugation, and maintenance in PBMC media: Stemspan H3000 (StemCell Technologies) supplemented with 50 ng/ml SCF, 40 ng/ml IGF-1 (Miltenyi), 10 ng/ml IL-3 (ThermoFisher), 2 U/ml EPO (R&D), 50 *μ*g/ml ascorbic acid, and 1 *μ*M dexamethasone (Sigma). Two million PBMCs were nucleofected with episomal reprogramming plasmids (Addgene 27080, 27078 and 27077), which expressed transcription factors L-MYC, LIN28, SOX2, KLF4, and OCT3/4, using the Amaxa-4D nucleofection system, Lonza P3 kit with 1 *μ*g total plasmid amount. Transfected cells were seeded on Geltrex-coated plates containing Essential 8 (E8) medium (Life Technologies) and maintained daily until identifiable hiPSC colonies were observed (2-3 weeks). Suitable hiPSCs were then manually picked and transferred to Geltrex-coated plates for expansion in the E8 medium. The control hiPSC line was derived from normal skin fibroblasts (European Collection of Authenticated Cell Cultures (ECACC)) by the pluripotent stem cell core facility, StemCore (University of Queensland, Australian Institute for Bioengineering and Nanotechnology, Brisbane, Australia).

### 2.5. Characterisation of hiPSC Lines: In Vitro Trilineage Differentiation and Assessment of Pluripotency

To characterise pluripotency, embryoid bodies (EBs) were formed by transferring iPSC aggregates to nontissue culture-treated dishes containing E8 medium and 10 *μ*M Y-27632 (Sigma). The next day, the medium was replaced with spontaneous differentiation medium: KnockOut Dulbecco's Modified Eagle's Medium, 20% KnockOut serum replacement, 1% nonessential amino acids, 1x penicillin-streptomycin, 1% glutamax, and 0.1 mM *β*-mercaptoethanol (Life Technologies). On day 7, EBs were plated onto Geltrex-coated wells and cultured a further 7 days in the spontaneous differentiation medium prior to cell harvest for RT-qPCR and immunofluorescence assessment of pluripotency.

To determine gene expression levels, we first extracted total RNA using the RNeasy micro kit (Qiagen, USA). RNA was then converted to cDNA using the SuperScript IV 1^st^-Strand Synthesis System kit (Invitrogen, USA). Relative gene expression was determined using 1x SensiMix SYBR (Bioline) and the Rotor-Gene 6000 Cycler system (Qiagen). Relative gene expression was calculated by 2^-*Δ*Ct^ analysis of housekeeping genes *POL2A* and *HPRT* against the lineage markers: *AFP*, *EN1*, *PAX6*, *CDH20*, *PHOX2B*, *FOXF1*, and *HAND2*. Statistical analysis was performed by unpaired *t*-test. Primers were designed using PrimerBlast and synthesised by Sigma (see Supplementary Table S1).

For immunofluorescence assessment of pluripotency, we used the StemLight™ iPS Cell Reprogramming Antibody Kit #9092 (Cell Signalling Technologies). In brief, cells cultured on glass coverslips were fixed in 4% paraformaldehyde/PBS for 10 min at room temperature and permeabilised with 1% Triton X-100/PBS for 5 min, then blocked with fish gelatin (Sigma) followed by primary antibody incubation overnight at 4°C. Secondary antibody and DAPI incubation occurred in the dark for 2 h. After PBS washing, slides were mounted in 70% glycerol/PBS and imaged by Axio Imager fluorescence microscopy (Zeiss).

### 2.6. hiPSC Genomic Characterisation via SNP Chromosome Microarray

SNP Chromosome microarray analysis was implemented to confirm genome integrity by examining genomic DNA extracted from hiPSC cells. The Illumina Human CytoSNP-12 assay (Illumina, USA) was performed according to manufacturer's protocols. In summary, 300 ng of hiPSC gDNA underwent whole-genome amplification overnight at 37°C, before fragmentation, purification, and loading onto a Human CytoSNP-12 BeadChip for overnight hybridisation at 48°C. The next day, the BeadChip underwent single base-pair extension and staining using the illumina Automation Control program (Illumina, USA) on the Tecan Freedom EVO liquid handler (Tecan, Switzerland). SNP microarray analysis was performed using BlueFuse Multi v4 (Illumina, USA), with the variant calling algorithm settings as follows: dosage log ratio quality metric (DLRDev) < 0.24, ≥8 consecutive adjacent SNP probes consistent with the copy number change detected, with LogR ratios being > +0.2 and < -0.3 for duplication and deletions, respectively.

### 2.7. Differentiation to hiPSC-RPE and Subsequent Characterisation

A stepwise protocol was used to differentiate hiPSCs into retinal pigmented epithelial cells as previously described with some modifications [12, 13]. In brief, hiPSCs cultures at 90% confluency were given Essential 6 medium (Life Technologies) for 2 days followed by 3-4 weeks of proneural induction media: advanced DMEM/F12, 1% glutamax, 1% nonessential amino acids, and 1x penicillin-streptomycin (Life technologies). Emerging pigmented islands of RPE appearing in differentiating cultures were then manually excised using a 21G needle and further cultured to form a monolayer of hiPSC-RPE cells maintained over a period of 90-120 days in spontaneous medium: KnockOut DMEM, 20% KnockOut serum replacement, 1% nonessential amino acids, 1x penicillin-streptomycin, 1% glutamax, and 0.1 mM *β*-mercaptoethanol (Life Technologies). Presence of RPE cells was confirmed using immunofluorescence and quantitative RT-PCR methods as above, with hiPSC-RPE cells stained with rabbit-anti-ZO-1 (1 : 200 dilution, Sigma) and mouse-anti-MitF (1 : 200, Exalpha) antibodies. Markers used for RT-qPCR studies were as follows: *BEST1*, *MERTK*, *MITF*, *PAX6*, *PMEL17*, and *RPE65*. Relative gene expression was calculated by 2^-*Δ*Ct^ analysis compared to the expression of housekeeping genes *POL2A* and *HPRT*. Statistical analysis was performed by unpaired *t*-test. Primers were designed using PrimerBlast and synthesised by Sigma (see Supplementary Table S1).

### 2.8. RNA Splicing Assay in hiPSC-RPE Cells

Total RNA was extracted from hiPSC-RPE cells lines using the RNeasy Micro kit (Qiagen, USA), according to manufacturer protocols. Total RNA was converted to cDNA using the SuperScript IV 1^st^-Strand Synthesis System kit (Invitrogen, USA). Primers were designed using Primer3 and had a forward primer specific for the 5′UTR of *RPE65*, with reverse primers specific to exon 4 and exon 5, respectively. PCR was performed using standard cycling conditions. Primers were synthesised by Sigma (see Supplementary Table S1). PCR products were resolved by agarose gel electrophoresis, with resultant bands excised and purified using the Wizard® SV Gel and PCR Clean-Up System (Promega, USA) before Sanger sequencing (Australian Genome Research Facility, Westmead NSW, Australia). An orthogonal *in vitro* assay to confirm potential aberrant splicing resulting from the novel *RPE65* genomic variant was undertaken using an ExonTrap vector minigene system (MoBiTech, GmbH, Germany) (see Supplementary File, Methods).

### 2.9. Western Blotting

Total protein was extracted from hiPSC-RPE cells using RIPA buffer (ThermoFisher) and 1x protease inhibitor (Roche). Protein concentration of cell lysates was determined using the Direct Detect Assay-free cards (MerkMillipore, Australia), and 30 *μ*g of protein from each lysate was resolved by 4-20% Tris-glycine SDS-PAGE (ThermoFisher, Australia). Protein was transferred onto a nitrocellulose membrane, which was blocked with 5% skim milk in 1x TBS and then incubated with rabbit anti-RPE65 (PA5-78414, ThermoFisher, USA) and mouse anti-*β*-actin (Sigma) antibodies, followed by fluorescent dye conjugated secondary antibodies (anti-rabbit IRDye 800CW and anti-mouse -IRDye 680RD, Millennium Science, Australia). Fluorescence detection was made using the ChemiDoc MP Imaging System (Bio-Rad, Australia), and acquired images were exported to Image Lab 6.0 software for quantification of RPE65 65 KDa protein band intensities normalised to *β*-actin levels (loading control).

## 3. Results

### 3.1. Ophthalmic Clinical Presentations

The two affected siblings in this study presented with nyctalopia in early childhood. Patient II-1 (Figure 1(k)), a female, presented at 4 years of age when her parents recognised her difficulty with night vision and adaptation to changing lighting conditions. Systemic assessment noted normal developmental milestones, normal hearing, normal bone development, and no family history of visual problems. Her best corrected visual acuity was 6/6 bilaterally. Her fundal appearance showed discrete RPE mottling in the midperiphery illustrating outer retinal atrophy and small white intraretinal spots in the perimacular region (Figures 1(a) and 1(b)). The international society for clinical visual electrophysiology (ISCEV) standard ffERG performed at 5 years of age showed undetectable scotopic responses in each eye, and the photopic responses were defined but reduced in each eye consistent with a rod-cone dystrophy (Figure 1(j) top row). A diagnosis of early-onset severe retinal dystrophy (EOSRD) was made. Repeat ffERG at 12 years of age identified further reduction in rod-cone function. A pattern electroretinogram (pERG) showed a normal response to the 15-degree field and reduced response to the 30-degree field in the right eye and reduced responses to both the 15- and 30-degree fields in the left eye. Wide-field fundus photographs attained at age 12 showed widespread distribution of retinal atrophy with associated white fleck-like lesions in the peripheral fundus. The WF-FAF was almost absent. SD-OCT assessment showed preservation of the ellipsoid zone and outer-retinal structures. (Figure 1(c)).

Patient II-2, a female, was noticed to have difficulty with night vision from the age of 2 years. There were no systemic medical associations. At age 5 years, she underwent a ffERG which showed an undetectable scotopic ERG and residual severely attenuated photopic ERG, consistent with an early onset severe retinal dystrophy in the (EOSRD) (Figure 1(j) middle row). Wide-field pseudocolor fundus imaging attained at age 5 showed widespread distribution of retinal atrophy with associated white fleck-like lesions in the peripheral fundus (Figure 1(d)). The WF-FAF was virtually absent (Figure 1(e)). SD-OCT showed preservation of the ellipsoid zone across the macula with slight macular thinning (Figure 1(f)).

Parental ophthalmic assessments including electrophysiology testing were entirely normal including pERG and ffERG WF-FAF and SD-OCT.

### 3.2. Genomics, Segregation Analysis, Bioinformatics, and Review of the Clinical Phenotype

To establish the genotypic mutation of the EOSRD diagnosis, targeted capture gene panel testing (Molecular Vision Laboratories, USA) was undertaken. Analysis of the proband II-1 was unable to identify a clear cut clinically significant genetic cause. Interestingly, the results showed two variants in the *RPE65* gene (NM_000329.2):c.93A>G;271C>T p.(Thr31Thr);(Arg91Trp) (Figure 1(k)). The presence of these individual variants was confirmed using Sanger sequencing in the proband and her affected sister. Segregation studies were consistent with autosomal recessive inheritance, with one of each of the variants present in each of the parents (Figure 1(k)). The *RPE65*:c.271C>T p.(Arg91Trp) variant was classified as pathogenic as it has been reported as disease causing in both compound heterozygous and homozygous states in patients with autosomal recessive LCA [14], EOSRD [15], and RP [16]. Functional studies using isomerohydrolase and protein stability assays of this variant showed a significant decrease in RPE65 enzymatic activity and protein level [17, 18]. However, the second heterozygous variant *RPE65*:c.93A>G p.(Thr31Thr) was a synonymous change which was novel and absent from population databases including gnomAD (version 2.1.1). We noted that this *RPE65*:c.93A>G variant affected the penultimate nucleotide to the canonical donor splice site of exon 2. Predictive *in silico* splicing tools using Alamut Visual v2.13 (Interactive-Biosoftware, France) and SpliceAI [19] indicated reduction in the splicing efficiency of the natural canonical splice donor site of exon 2. These findings prompted review of the retinal phenotype in both siblings, and it was noted that peripheral retinal white flecks and reduced fundus autofluorescence would be consistent with *RPE65*-associated retinopathy, providing supporting evidence to further investigate the functional impact of the *RPE65*:c.93A>G p.(Thr31Thr) synonymous variant [20, 21].

### 3.3. Characterisation of hiPSC Lines and Differentiation to hiPSC-RPE

**Figure 2:**
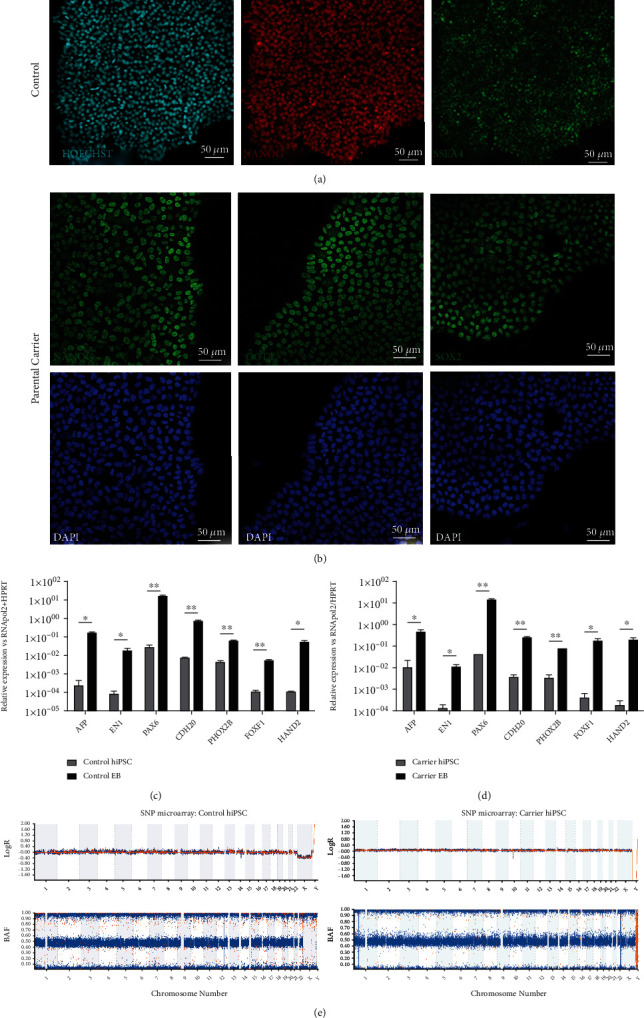
Characterisation of control and parental carrier (c.93A>G) hiPSC lines derived from human somatic cells. (a, b) Immunofluorescence detection of pluripotency markers NANOG, SSEA-4, OCT4, and SOX2 expressed in the nuclei of the control and carrier (c.93A>G) hiPSC lines. Nuclei stained with either Hoechst (cyan) or DAPI (blue), scale bar: 50 *μ*m. (c, d) Verification of trilineage differentiation capacity of the control and carrier hiPSCs by RT-qPCR detection of increased relative gene expression of ectoderm (*EN1*, *PAX6*), endoderm (*AFP*, *CDH20*, and *PHOX2B*), and mesoderm (*FOXF1*, *HAND2*) markers in differentiated EBs compared to hiPSCs from both lines (unpaired *t*-test, *n* = 3 replicates, ^∗^*P* < 0.05; ^∗∗^*P* < 0.005). (e) SNP chromosome microarray analysis showing genome integrity and normal karyotype for the control and carrier hiPSCs.

To investigate the predicted splicing defect, we generated two hiPSC lines, one from a healthy control, and the other from the heterozygous parental carrier of the novel *RPE65*:c.93A>G variant. Immunocytochemistry of both hiPSC lines demonstrated presence of OCT4, NANOG, and SOX2/SSEA-4 pluripotency markers (Figures 2(a) and 2(b)). Pluripotency was also demonstrated by increased transcript expression of genes for each lineage (ectoderm, endoderm, and mesoderm) after 2 weeks of *in vitro* trilineage differentiation (Figures 2(c) and 2(d)). Digital karyotyping using SNP chromosome microarray confirmed that both control and c.93A>G carrier hiPSCs had normal karyotypes (46,XY and 46,XX, respectively) (Figure 2(e)).

**Figure 3:**
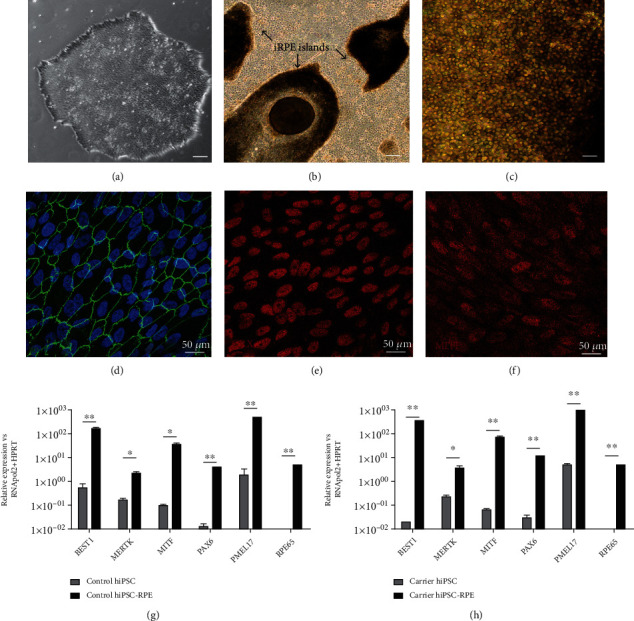
Differentiation of hiPSCs to hiPSC-RPE cells. (a) Representative brightfield image of a parental carrier hiPSC colony, scale bar = 100 *μ*m. (b) Formation of pigmented RPE islands in day 30-40 retinal differentiation cultures, scale bar = 100 *μ*m. (c) Cell culture expansion of RPE islands formed a purified monolayer of hiPSC-RPE cells after 90 days of differentiation, scale bar = 100 *μ*m. (d) hiPSC-RPE cells cultured on coverslips were fixed in 4% paraformaldehyde/PBS and stained with ZO-1 (green) tight junction expression highlighting the hexagonal shape of hiPSC-RPE cells. Nuclei stained with DAPI (blue). (e, f) Immunofluorescence detection of RPE markers CRX and MITF (red) in hiPSC-RPE cells. (g, h) Verification of differentiation of control and carrier (c.93A>G) hiPSCs to RPE cells by RT-qPCR detection of increased relative gene expression of RPE markers (*BEST1*, *MERTK*, *MITF*, *PAX6*, *PMEL17*, and *RPE65*) (unpaired *t*-test, *n* = 3 replicates, ^∗^*P* < 0.05; ^∗∗^*P* < 0.005).

We next sought to produce retinal-specific tissue and perform functional evaluation by differentiating both hiPSC lines into RPE cells. Monolayers of day 90-120 hiPSC-RPE cells showed clear pigmentation and typical cobblestone-like morphology characteristic of human RPE cells (Figures 3(a)-3(c)). ZO-1 staining identified tight junctions expressed throughout hiPSC-RPE monolayers (Figure 3(d)) together with the expression of RPE markers CRX and MITF (Figures 3(e) and 3(f)). Gene expression analysis of other RPE-specific markers *BEST1*, *MERTK*, *MITF*, *PAX6*, *PMEL17*, and *RPE65* showed increased expression in both the control and carrier hiPSC-RPE cells (Figures 3(g) and 3(h)).

### 3.4. RNA Studies of Synonymous Variant in hiPSC-RPE Cells Identify Exon Skipping Leading to Reduced RPE65 Protein Expression

**Figure 4:**
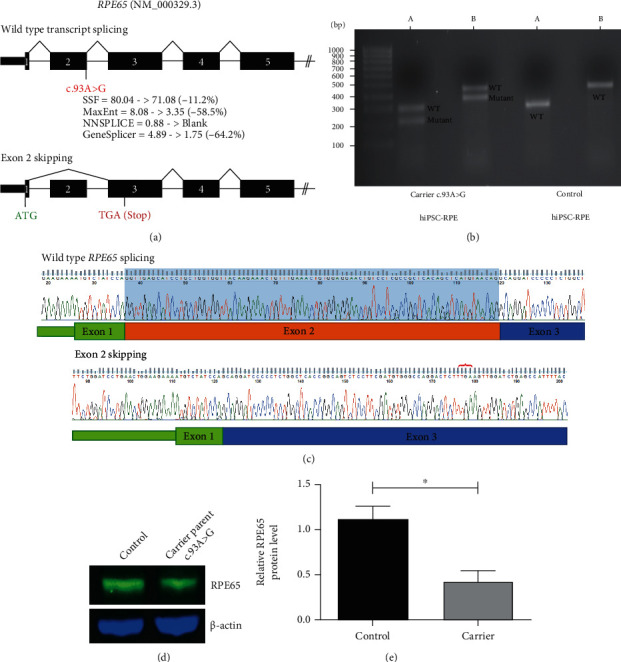
RNA and protein studies of the *RPE65* c.93A>G variant. (a) Schematic of wild-type and mutant transcripts and location of the novel c.93A>G variant are shown. Predicted reduction in splice donor strength due to this variant using Alamut Visual is annotated beneath. The site of the predicted premature stop codon (TGA) is indicated in red on the mutant transcript schematic. (b) Representative agarose gel image of cDNA studies illustrating additional presence of a smaller band consistent with exon skipping in c.93A>G carrier hiPSC-RPE cells compared with control. A: amplicon primers located in 5′UTR and exon 4 (mutant allele = 215 bp; wild-type allele = 297 bp); B: amplicon primers located in 5′UTR and exon 5 (mutant allele = 368 bp; wild-type allele = 450 bp). (c) Purified gel band sequencing of the control and mutant cDNA sequence shows excision of exon 2 from the mutant transcript. Red bracket indicates the location of the premature stop codon introduced. Blue shading indicates normal Exon 2 sequence which is absent from the mutant transcript. (d) Western blot analysis showing RPE65 levels in the control [Left] and parental carrier hiPSC-RPE cells [Right]. (e). Density analysis of RPE65 protein bands shows approximately 50% reduction in RPE65 expression in the parental carrier hiPSC-RPE cells compared with the control (unpaired *t*-test, *n* = 3 independent experiments, ^∗^*P* < 0.05).

We next used the differentiated hiPSC-RPE cells to test whether the *RPE65*:c.93A>G variant was interfering with donor splice site activity at the exon 2-intron 2 boundary. The removal of exon 2 from the NM_000329.2 *RPE65* transcript is predicted to result in the introduction of a premature termination codon resulting in a mutant *RPE65* allele of only 22 amino acids in length (Figure 4(a)). RNA from hiPSC-RPE cells from the control and the carrier parent was extracted and RT-PCR performed. To ensure the PCR amplicons generated encompassed the splice site predicted to be affected, we designed *RPE65* 5′UTR primers and reverse primers specific to exons 4 and 5 (Figure 4(b), denoted as A and B, respectively). Wild-type and mutant bands were visible on agarose gel electrophoresis (Figure 4(b)), and cDNA sequencing of these products showed skipping of exon 2 in the mutant transcript (Figure 4(c)).

Further confirmation of exon 2 skipping of the novel synonymous variant was investigated using a minigene vector-based approach (see Supplementary File, Figure S1).

To investigate whether the aberrant splicing and predicted premature termination codon resulted in differences in RPE65 protein levels, we examined hiPSC-RPE cell lysates by western blot analysis and found an approximate 50% decrease in RPE65 in the parental carrier *RPE65*:c.93A>G RPE compared with control RPE (Figures 4(d) and 4(e); *n* = 3 independent experiments).

Variant details were submitted to the ClinVar database with accession details: SCV001905505.

## 4. Discussion

In this study, we demonstrate the value of parental stem cell-derived retinal tissue for genetic variant classification. The siblings studied here had a severe retinal dystrophy phenotype and review of genotypic findings and segregation studies showed compound heterozygous variants in *RPE*65, with one classified as a pathogenic variant and the other a novel synonymous VUS, *RPE*65:c.93A>G p.(Thr31Thr). Ocular phenotypic review indicated features including peripheral retinal white flecks and reduced fundus autofluorescence which could be consistent with *RPE*65-related disease. Bioinformatics analysis of the synonymous variant suggested a splicing abnormality, requiring functional studies of RNA expression to confirm this. As *RPE65* expression is limited to the clinically inaccessible RPE cells in the eye, we generated a parental-derived hiPSC line which in turn was differentiated to generate hiPSC-RPE cells carrying the synonymous variant. We demonstrated the skipping of exon 2 and change in reading frame in the *RPE65* coding sequence, which resulted in a predicted severely truncated mutant allele of only 22 amino acids. Confirming the predicted protein truncation, examination of the parental carrier hiPSC-RPE cells showed a reduction in *RPE65* protein expression. These findings were validated by an orthogonal ExonTrap minigene assay also demonstrating skipping of exon 2. The *RPE65*:c.93A>G p.(Thr31Thr) variant is a novel synonymous variant in *RPE65* we have shown to be of pathological significance. These results indicate the presence of biallelic pathological variants in these patients, enabling eligibility for *RPE65* gene replacement therapy.

The *RPE65* gene encodes the catalytic enzyme retinal isomerase, which plays a critical role in maintaining the presence of 11-cis retinol in the visual cycle [3]. There are >120 disease-causing *RPE65* variants reported in public online databases which have been associated with photoreceptor dysfunction and structural abnormalities involving the retina {https://www.ncbi.nlm.nih.gov/clinvar/RPE65; https://databases.lovd.nl/shared/genes/RPE65}. The majority of reported *RPE65* disease-causing alleles are missense variants (49%), followed by truncating variants (36%), splice site variants (12%), and in-frame deletions (3%) [4]. Investigation using hiPSC technology and derivative tissues such as RPE cells could delineate the molecular aetiology for other known *RPE65* variants. Interestingly, to date, there are no disease-associated splicing variants described resulting in the skipping of exon 2. There is, however, a recent report of a disease-associated nucleotide substitution implicating the final coding base of exon 2, namely *RPE65*:c.94G>T p.(Gly32Cys) [7, 22]. From the current literature, the deleterious effect of this particular variant is unclear as to whether it is due to the amino acid substitution (which has a codon across the splice site junction), or is reflective of a defect in natural canonical splicing. Given that both the c.93A>G described in this study and the c.94G>T variant are near the 5′ donor splice site, it is likely that the c.94G>T variant may also influence the efficiency of natural canonical splicing at this location. Studies such as the one described here would help clarify this question. Future experiments using our hiPSC-RPE model could also be effective in measuring retinoid isomerohydrolase activity to demonstrate the effect of a 50% reduction in functional *RPE65* protein, compared to a wild-type control.

Previous work involving another retinal-specific disease gene, *BEST1*, highlighted the value of patient-derived hiPSC-RPE for investigation of variants thought to potentially affect RNA splicing in the RPE, compared with assays in HEK293 cells where artefactual results arose [23]. These results suggested there may be differences in gene splicing of transcripts between RPE and non-RPE cells, as has been identified for some transcripts in photoreceptor cells compared with nonretinal tissues [24]. To ensure that splice assay results in this project were specific to RPE cells, we proceeded with hiPSC production and RPE cell derivation from a sample available from the carrier parent in this family. This demonstrated a splicing abnormality, which was also able to be confirmed in our minigene assay undertaken in a HEK293 model system. Recognised differences in splicing in retinal tissues compared with nonretinal tissues highlight the value of hiPSC-retinal derived cells to ensure results specific to the retinal tissue.

The recent emergence of gene therapies heralds a new era of treatment options for the retinal dystrophies. *RPE65* gene augmentation is the first gene replacement therapy for retinal disease in clinical use and is a landmark step in genomic and precision medical advancement. A key requirement for access to this therapy is monogenic disease with pathological, biallelic mutations as determined by diagnostic genomic testing [8]. Diagnostic laboratories currently follow strict guidelines for classifying genomic variants [9, 10]. Using this systematic approach, curation of novel variants is often challenging, especially with patients needing to meet strict inclusion criteria for *RPE65* gene augmentation therapy. The valuable contributions of modelling variants in iPSC-derived retinal cells is evident in emerging studies of frequently described IRD disease genes such as *RPGR* [11] and *USH2A* [25]. The hiPSC-derived cells in this study were critical for determining the pathological significance of a synonymous variant in *RPE65* and clearly demonstrated the value of a stem cell-based approach.

## 5. Conclusions

We generated a carrier parent-derived hiPSC-RPE model to establish the molecular pathogenesis of a novel synonymous genomic variant *RPE65*:c.93A>G p.(Thr31Thr), originally classified as a VUS on diagnostic genomic testing. RNA-based studies confirmed an aberration in gene splicing and generation of a loss-of-function allele with reduction of RPE65 protein expression. This study highlights the power of hiPSC technologies to establish the pathogenicity of genomic variants in disease genes with tissue-specific expression and showcases the collaborative opportunities between diagnostic laboratories and stem cell medical research to ensure positive outcomes for patients in the era of gene therapies.

## Data Availability

The data supporting the findings of this study are available upon request to the corresponding author. The genomic variant has also been submitted to the ClinVar repository.

## Supplementary Materials

Supplementary Materials Supplementary Methodology for the pET-01 ExonTrap minigene assay. Supplementary Figure S1: illustrating ExonTrap minigene findings. Supplementary Table S1: PCR primer sequences.
