# Supplementary material for: Evaluation for Retinal Therapy for RPE65 Variation Assessed in hiPSC Retinal Pigment Epithelial Cells
Source: Stem Cells Int. 2021 Dec 13;2021:4536382. doi: 10.1155/2021/4536382 (PMC8687838; doi:10.1155/2021/4536382)
Supplement: Supplementary Materials — Supplementary Methodology for the pET-01 ExonTrap minigene assay. Supplementary Figure S1: illustrating ExonTrap minigene findings. Supplementary Table S1: PCR primer sequences. [file 4536382.f1.docx]

**Supplementary Materials**

Supplementary Method

*pET-01 ExonTrap minigene assay*

As an orthogonal *in vitro* assay to investigate potential aberrant splicing resulting from the novel *RPE65* genomic variant, an ExonTrap vector minigene system (MoBiTech, GmbH, Germany) was applied. Briefly, we captured a 2.8Kb genomic DNA fragment containing exons 2 and 3 of the *RPE65* gene from a known heterozygous carrier of the variant using Q5 High-Fidelity Polymerase PCR (New England Biolabs, Australia) according to manufacturer recommendations. Genomic DNA primers were designed using Primer3 and were located >500bp upstream of the exon 2 and downstream of the exon 3 intron/exon boundaries of genomic RPE65 sequence, and included Sal1 and Not1 restriction site sequences to facilitate directional cloning into the ExonTrap minigene vector multi-cloning site (MCS). The integrity of the cloned gDNA segment in the ExonTrap minigene vector was verified by Sanger sequencing. Confirmed mutant and control plasmids were then transfected into HEK293 cells (DMEM + 10% FCS) using Lipofectamine 3000 (Invitrogen, USA). After 3 days, transfected cells underwent total RNA extraction and conversion to cDNA for RT-qPCR analysis using primers binding to the vector 5’ and 3’ exons, flanking the captured genomic fragment cloned into the ExonTrap vector. PCR amplicons generated from the mutant and control ExonTrap cDNA were purified using the Wizard® SV Gel and PCR Clean-Up System (Promega, USA) for Sanger sequencing (Macrogen, South Korea).

Supplementary Figure


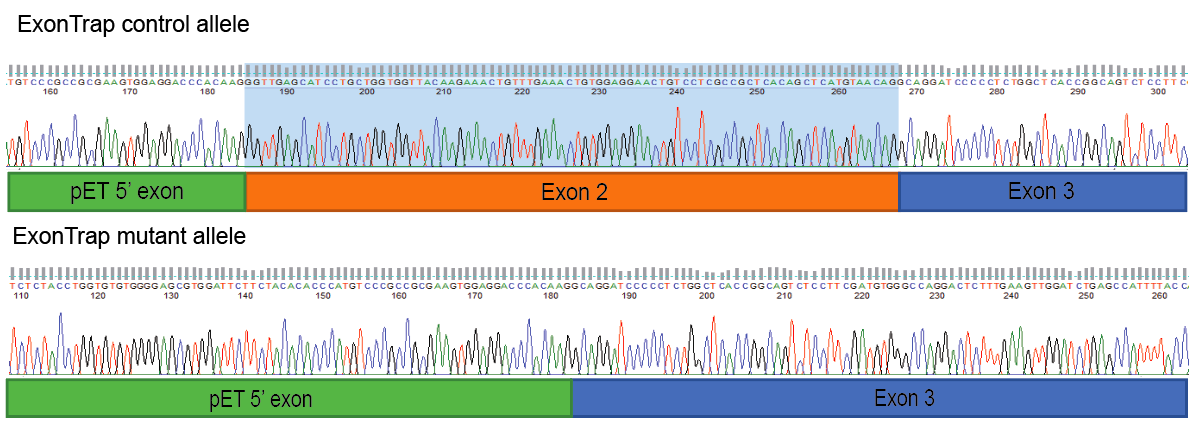


**Figure S1.** *ExonTrap minigene studies.* Transfection of both wildtype and mutant ExonTrap plasmids into HEK293 cells was followed by RNA extraction, cDNA synthesis and sequencing of *RPE65* gene fragments expressed by the minigene vector confirmed the excision of exon 2 sequence from RNA in the presence of the *RPE65*:c.93A>G variant. Wild-type and mutant cDNA sequences obtained from the ExonTrap minigene constructs are shown, confirming independently the removal of exon 2.

**Table S1:** List of PCR primer sequences used in this study.

| **Name** | **Sequence (5’-3’)** |
| --- | --- |
| *POLR2A* | F: GTGCGGCTGCTTCCATAA  R: GCACCACGTCCAATGACAT |
| *HPRT* | F: GACCAGTCAACAGGGGACAT  R: CCTGACCAAGGAAAGCAAAG |
| *AFP* | F: TGAGCACTGTTGCAGAGGAG  R: GTTCCAGCGTGGTCAGTTTG |
| *EN1* | F: CGTGGTCAAAACTGACTCGC  R: CGCTTGTCCTCCTTCTCGTT |
| *PAX6* | F: GAACAGACACAGCCCTCACA  R: ATAACTCCGCCCATTCACCG |
| *CDH20* | F:TGATAACCCACCCCGCTTTC  R: AAGGCATCTGCACCATCTCC |
| *PHOX2B* | F: CATCTACACTCGGGAGGAGC  R: CCTCTTTGCTCTCGTCGTCC |
| *FOXF1* | F: TGCACCAGAACAGCCACAA  R: TGCTGGTGGTAGTAGGAGCC |
| *HAND2* | F: CCAGCTACATCGCCTACCTC  R: CCGGCCTTTGGTTTTCTTGT |
| *BEST1* | F: TATCCTGCTCCAGAGCCTGC  R: GAAGCTGTACACCGCCACA |
| *MERTK* | F: TGGGGTCCAGAACCATGAGA  R: GAAAAGGTGGGGCGGTCTAA |
| *MITF* | F: AAGCAAGAGCACTGGCCAAA  R: ATGGTTCCCTTGTTCCAGCG |
| *PMEL17* | F: TGATAGGTGCTTTGCTGGCT  R: CTGGGCTTCTGTCCACTCTG |
| *RPE65* | F: GCCGCTCACAGCTCATGTAA  R: GCTTGCCCATCAAACAGGTG |
| *RPE65* 5’UTR-Ex4 | F: tctggatcctgaactggaaga  R: gcccgtacgtaagcatcagt |
| *RPE65* 5’UTR-Ex5 | F: tctggatcctgaactggaaga  R: aatcttcccccactgggtag |

F: Forward, R: Reverse
